# Intestinal Protective Effects of Herbal-Based Formulations in Rats against Neomycin Insult

**DOI:** 10.1155/2013/161278

**Published:** 2013-04-11

**Authors:** Shambhunath Bose, Kyung-Wan Han, Myeong-Jong Lee, Hojun Kim

**Affiliations:** ^1^Institute of Medical Research, College of Medicine, Dongguk University-Seoul, Goyang 410-773, Republic of Korea; ^2^College of Pharmacy, Dongguk University-Seoul, Goyang 410-820, Republic of Korea; ^3^Department of Oriental Rehabilitation Medicine, College of Oriental Medicine, Dongguk University Ilsan Hospital, 814 Siksa-dong, Gyeonggi-do, Goyang 410-773, Republic of Korea

## Abstract

Disturbance in the gut microbial niche by antibiotics like neomycin produces gastrointestinal (GI) disorders. Here, we evaluated the impact of a mixture of extracts of three herbs (Atractylodis Rhizoma Macrocephalae, Massa Medicata Fermentata, and Dolichoris Semen) with known GI protective activities, either laboratory unfermented (herbal formulation-1 (HF-1)) or fermented/re-fermented (herbal formulation-2 (HF-2)) on neomycin-treated rats using a commercial *Lactobacillus* probiotic as a reference. Treatment with neomycin augmented stool water content, decreased fecal population of *Lactobacillus* spp., changed the histology of intestine without inducing inflammation, reduced the colonic expression of zonula occludens-1 (ZO-1) and claudin-1, and elevated the serum C-reactive protein (CRP) and interferon-gamma (IFN-**γ**) levels. Coadministration of either HF-2 or probiotic, but not HF-1, restored the fecal content of *Lactobacillus* spp., normalized the serum CRP level, and significantly increased the colonic expression of ZO-1 and claudin-1 in neomycin-treated rats. The combined treatment with any of the above agents ameliorated the histological changes of cecum and colon in neomycin-treated rats, and the magnitude of this effect was probiotic > HF-2 > HF-1. Our study revealed the intestinal protective effect of a mixture of three herbs against neomycin insult, which is mediated through multiple mechanisms and is potentiated upon prior fermentation/refermentation of the herbs.

## 1. Introduction 

The mammalian system is colonized by trillions of microbes, the majority of which live in gastrointestinal (GIT) tract, predominantly by maintaining a symbiotic relationship with their host. The gut commensal bacteria influence the health of their host by exerting effects on a number of parameters [1], and substantial evidence indicates that microbiota modulates a series of events at both the cellular and molecular levels that are crucial for maturation, differentiation, and proliferation of the intestinal membrane (IM) as well as maintaining the integrity of barrier function [2]. In a healthy mammalian system, the gut epithelial barrier function and permeability are regulated by the apical junctional complex which is constituted by tight junction proteins, like those belonging to occludin, claudin, and zonula occludens families [3]. Microbial colonization of GIT plays an important role in the protection of the epithelial barrier by maintaining expression of the tight junction proteins [2, 4].

In mammals, antibiotic treatment is often associated with long-term decrease in beneficial microorganisms and augmentation of potentially harmful microbes [5]. Neomycin, a nonabsorbable, broad-spectrum antibiotic commonly used in sterilizing the GIT, reduces the population of aerobic intestinal bacteria [6]. Destruction or disturbance in gut microbial homeostasis by antibiotics weakens the intestinal barrier, ultimately leading to increased intestinal permeability [4]. Additionally, neomycin can also produce a number of adverse effects on the physiology, morphology, and histology of the GIT [7–10], the factors that also collectively contribute to the destabilization of intestinal barrier integrity. As a consequence of increased intestinal membrane (IM) permeability, the possibility of translocation of viable indigenous microbes from GIT to extraintestinal sites could be enhanced, which in turn may cause the induction of a number of diseased states and pathogenesis. Indeed, antibiotic-mediated perturbation of the intestinal microbiota is responsible for changing the host susceptibility to enteric infection [11] which may lead to diarrhea [12].

Substantial evidence has indicated the implication of complementary and alternative medicines in the treatment of GIT diseases [13–16], among which many are used as dietary herbs. Accordingly, the dried rhizome of Atractylodis Rhizoma Macrocephalae (ARM, also known as Bai Zhu), Massa Medicata Fermentata (MMF), and Dolichoris Semen (DS), which are also employed in different dietary preparations in Asian countries, are being frequently used in various herbal formulations for the treatment of a number of GIT disorders in humans and animals [13, 14, 16].

The present study was conducted to evaluate the beneficial effect and mode of action of mixed extracts of the above three herbs on the GIT of rats challenged with neomycin. A number of herbal formulas of traditional Japanese medicines (Kampo) as well as Chinese and Korean ones utilize mixtures of several herbs (multiherbs) in a single formula [13, 14, 16]. On the other hand, as the beneficial health effects of probiotics and their fermented food products are well known [27] including the prevention of diarrhea caused by antibiotics [28], we also used the mixed fermented/refermented extracts of the above three herbs in our experiment to judge whether our laboratory-fermented formulation in association with the probiotics employed would be advantageous over the corresponding laboratory-unfermented preparation in combating the adverse impact of neomycin. For this purpose, *Leuconostoc mesenteroides* was employed for the refermentation of MMF, whereas *Bacillus licheniformis* was used for the fermentation of both ARM and DS. *Leuconostoc *spp. play an important role in the fermentation of various food products including vegetables like sauerkraut, kimchi, pickles, and sayur-asin, and are also used as probiotics [29]. On the other hand, *B. licheniformis*, which is listed in the Third Edition of *The Food Chemicals Codex* (1981) as a source of carbohydrase and protease enzyme preparations, has been safely used for large-scale industrial fermentations as well as in commercial probiotics products for human and animal use [30, 31]. Finally, the GIT protective efficacy of the aforementioned herbal preparations was compared with that of a commercial *Lactobacillus acidophilus* probiotics being used as a reference.

## 2. Methods and Materials

### 2.1. Herbal Extraction and Fermentation/Refermentation

The extraction and fermentation/refermentation of the herbs were performed following our laboratory optimized procedures (Table 1). Briefly, the extract of individual herb was prepared by boiling the raw herb at 100°C for 2 h. The decocted herbal preparation was then subjected to evaporation and freeze-drying to produce the dried extract (yield approximately 10%). For the animal experiments, 20 g of the dried extract of each raw herb was mixed with 200 mL of boiled Milli-Q water, subjected to ultrasonication at 70°C for complete dispersion, and then incubated at 70°C for 3 h in a water bath under continuous shaking. Following this, the samples were either supplemented with glucose (2% w/v, for MMF and ARM) or the Luria-Bertani (LB) broth powder (2.5% w/v, for DS). All preparations were then autoclaved for 20 min at 121°C, which in addition to sterilization of the samples and killing the microbes involved in the natural fermentation of the MMF also served to further decoct the herbal products. After cooling the extracts to room temperature, the samples dedicated for fermentation/refermentation were inoculated with fresh subculture (2% v/v) of bacteria (*L. mesenteroides* for refermentation of MMF and *B. licheniformis* for the fermentation of both ARM and DS) and fermented for 24 h either at 35.4°C (*L. mesenteroides*) or at 31°C (*B. licheniformis*). The selection of the above herb-specific bacterial strains and incubation temperature was based on the optimization of the fermentation process performed in our previous study [32]. The corresponding unfermented samples were prepared in a similar manner, except for that they received 2% (v/v) of the respective sterile bacterial culture medium instead of the bacterial inoculum. Finally, the corresponding laboratory-unfermented or -fermented/refermented herbal extracts were combined together in equal volumes, mixed vigorously, and then subjected to low speed centrifugation. The supernatant portions of the resultant mixed extracts (HF-1 and HF-2, resp.) were stored at −70°C until used for oral dosing of the animals.

### 2.2. Determination of Total Polyphenol Content of the Herbal Preparations

Total polyphenol content of the herbal preparations was measured following the Folin-Denis colorimetric method [33] with some modification. Briefly, 25 *μ*L of each herbal preparation was added to 775 *μ*L water in microcentrifuge tubes and mixed thoroughly. To this mixture, 50 *μ*L of the Folin-Denis reagent (Sigma-Aldrich, St. Louis, MO, USA) was added and mixed vigorously. After one minute, 150 *μ*L of 20% sodium carbonate solution was added, and the contents were mixed thoroughly. The reaction mixture was then incubated in dark for 1 h at room temperature. Following this, the tubes were centrifuged for 5 min at 3000 rpm. An aliquot of the resultant supernatant was transferred to the individual well of a 96 well microtiter plate, and the absorbance was read at 750 nm using a microplate reader (Spectramax Plus, Molecular Devices, Sunnyvale, CA, USA). A calibration curve was prepared using gallic acid (Sigma-Aldrich) as a standard which was used further for determining total polyphenol in the samples. The data were expressed as mg gallic acid equivalent (GAE) per g of the herbal extract.

### 2.3. Animals and Treatment

Male 8-week-old Sprague-Dawley rats (Orient Bio, Seongnam-si, Republic of Korea) weighing 200 ± 20 g were housed in controlled conditions of temperature (20 ± 2°C), relative humidity (40%–60%), and a 12 h light-dark cycle (lights on at 7:00 Am). The animals were given access to standard normal chow diet (Soya Greentec, Hwaseong-Si, Republic of Korea) containing 20% protein, 4.5% fat, 63% calories from carbohydrate, and water *ad libitum*. All experimental procedures, including the care and handling of animals, were performed following the international guidelines [34]. The rationale, design, and protocols of this study were approved by the Institutional Animal Ethical Committee, Dongguk University. After acclimatization for 7 days, the animals were randomly divided into different experimental groups as follows: (1) control; (2) neomycin; (3) neomycin + HF-1; (4) neomycin + HF-2; (5) neomycin + probiotic. The neomycin (Calbiochem/EMD Biosciences, La Jolla, CA, USA) was dissolved in sterile water and administered orally to the animals in groups 2–5 at a dose of 1000 mg/kg, once daily for 7 consecutive days, while group 1 received sterile water only. The rats in groups 3 and 4 received oral administration of HF-1 and HF-2 formulations, respectively, at a volume (per kg body weight basis) that represented 200 mg of decoction extracted product of each raw herb. The dosing was performed once daily for 8 consecutive days, starting one day before the first dose of neomycin. The herbal dose was selected on the basis of the upper limit of recommended dose of raw herbs (20 g/day) in the traditional medical practices (for decocted products) for an adult human (60 kg body weight) [35]. This is equivalent to the daily oral dose of 205.5 mg of decocted product of each raw herb used in our study per kg body weight in rat (considering 10% yield in the decoction of raw herbs as estimated in our experiment), approaching very near to our experimental dose. 

The following calculation was applied for the conversion of adult human dose to rat dose.

Human equivalent dose (mg/kg) = rat dose (mg/kg) × (rat *K*_*m*_/human *K*_*m*_), where the body weight of adult human is considered as 60 kg and *K*_*m*_ values for rat and adult human are considered as 6 and 37, respectively.

Instead of herbal formulations, the animals in groups 2 and 5 were fed with water and probiotic (containing *L. acidophilus*, 1.0 × 10^11^ CFU/g, Cell Biotech, Gimpo-Si, Gyeonggi-do, Republic of Korea; dose: 0.16 g/kg body weight), respectively, as per the above schedule. Following the treatment regimen, the rats were anesthetized, and blood was collected by cardiac puncture. The intestine was surgically removed for further processing, and the feces were collected. Serum was obtained by centrifuging the blood at 1000 ×g for 15 min at 4°C. 

### 2.4. Determination of Fecal Water Content

Following collection, the stool of each rat was weighed rapidly and recorded as wet weight. The stool samples were then subjected to centrifugal evaporation for 2 h and weighed as dry weight. The water content of the stool was calculated according to the following formula: Water content (%) = (wet weight (g) − dry weight (g)/wet weight (g)) × 100.

### 2.5. Measurement of Serum CRP and IFN-*γ*

The serum CRP and IFN-*γ* levels were measured by ELISA using rat-specific commercial kits from BD Biosciences (San Diego, CA, USA) and Thermo Scientific (Rockford, IL, USA), respectively. The assays were performed following the instructions of the kit manufacturers.

### 2.6. Determination of *Lactobacillus* spp. and Universal Bacterial DNA Content in Rat Stool by Quantitative Real-Time PCR (qRT-PCR)

DNA was extracted from the stool by using a DNA Stool Mini Kit (Qiagen, Valencia, CA, USA) following the instructions of the kit manufacturer. The purity and concentrations of DNA in the samples were determined by spectrophotometry. The qRT-PCR of the samples was conducted in a LightCycler instrument (Roche Applied Science, Indianapolis, ID, USA) using a LightCycler FastStart DNA Master SYBR Green kit (Roche Applied Science). The amplification reactions were carried out following the instructions of kit manufacturer in a total reaction volume of 20 *μ*L containing PCR mix, template DNA (100 ng), primers (10 pmol for each), and bovine serum albumin (2.1 *μ*g). The sequences of the primers (Bioneer, Daejeon, Republic of Korea) targeting the 16S rRNA gene of the universal bacteria or *Lactobacillus* spp. are depicted in Table 2. PCR amplification conditions were a prior incubation step at 95°C for 10 min followed by 40 cycles of amplification encompassing denaturation at 95°C (10 s, for universal bacteria and 15 s for *Lactobacillus* spp.), annealing at 60°C (10 s for universal bacteria and 20 s for *Lactobacillus* spp.), and extension at 72°C (15 s, for universal bacteria and 45 s for *Lactobacillus* spp.). This was followed by melting curve analysis to verify the specificity of the amplicon. The resultant data were analyzed using the dedicated LightCycler software provided by the instrument manufacturer (Roche Applied Science). DNA levels were approximated 2^−*C*_*t*_^, where *C*_*t*_ is the crossing threshold value calculated by the software. The abundance of *Lactobacillus* spp. in the samples was calculated relatively as the ratio of 2^−*C*_*t*_^ of *Lactobacillus *spp. to that of universal bacteria. 

### 2.7. Determination of Colonic Gene Expression of Rat by qRT-PCR

The total RNA from the collected colon tissues was prepared using an RNeasy Mini Kit (Qiagen) in accordance with the kit manufacturer's instructions. An equal amount of RNA (1 *μ*g) from the samples was reverse transcribed to produce first strand cDNA using a Sprint RT Complete Oligo-(dT)_18_ cDNA synthesis kit (Clontech, Mountain View, CA, USA) following the instructions of kit manufacturer. qRT-PCR of the DNA samples was carried out as stated above for the stool microbial DNA in a final reaction volume of 20 *μ*L containing PCR mix, 1 *μ*L of DNA, and gene specific primers (10 pmol for each, Table 3). PCR amplification conditions were a prior incubation step at 95°C for 10 min followed by 40 cycles of amplification encompassing denaturation at 95°C for 10 s, annealing at the corresponding optimized temperature for 10 s, and extension at 72°C for 15 s. This was followed by melting curve analysis to verify the specificity of the amplicon. The quantification of relative gene expression was represented by standard 2^−Δ*C*_*t*_^ calculations using the housekeeping gene glyceraldehyde 3-phosphate dehydrogenase (GAPDH) for normalization, where Δ*C*_*t*_ =  (*C*_*t*-target  gene_ gene − *C*_*t*-GAPDH_).

### 2.8. Histology

Tissue sections (4 *μ*M in thickness) prepared from 10% buffered formalin-fixed and paraffin-embedded cecum and colon were mounted onto slides, stained with hematoxylin and eosin, and observed on a microscope (Olympus BX61, Tokyo, Japan). The images were captured with an Olympus DP70 digital camera.

### 2.9. Statistical Analyses

The values are expressed as means ± SEM. The statistical package for social science (SPSS) software program (version 17.0; SPSS, Chicago, IL, USA) was applied for analyses of the data. One-way ANOVA followed by Bonferroni's post hoc test was employed for the determination of significant differences between the study groups of the animals. Post hoc analyses were performed only when the means were significantly different in one-way ANOVA. When the error variance was found to be heterogeneous using Levene's test, logarithmic transformation of raw data was performed and indicated accordingly. Independent sample *t*-test was carried out to determine the significant difference in the polyphenol content between the unfermented and fermented/refermented preparations of MMF, ARA, DS, and mixed herbs. Differences were considered significant at *P* < 0.05. 

## 3. Results and Discussion

### 3.1. Polyphenol Content of the Herbal Preparations

**Figure 1:**
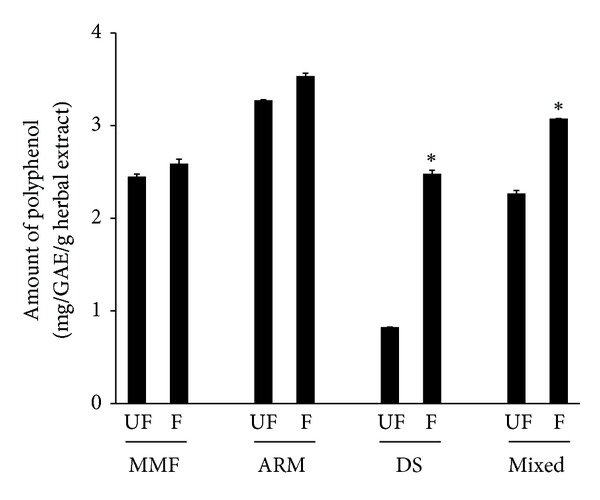
The total polyphenol content of unfermented (UF) and fermented (F) preparations of Massa Medicata Fermentata (MMF), Atractylodis Rhizoma Macrocephalae (ARM), Dolichoris Semen (DS), and mixed herbs. The detailed experimental conditions are described in Section 2. Values are means ± SEM, *n* = 3. * Significantly different from the corresponding unfermented preparation (*P* < 0.05).

Following fermentation, an increase in the total polyphenol was seen in all herbal preparations, although this change was found to be insignificant for both MMF and ARM (Figure 1). While the polyphenol content of DS was significantly elevated (3.03-fold, *P* < 0.05) because of fermentation. On the other hand, the total polyphenol of HF-2 preparation was significantly higher (1.36-fold, *P* < 0.05) compared to HF-1 formulation. 

### 3.2. Assessment of the Body Weight of Animals

**Figure 2:**
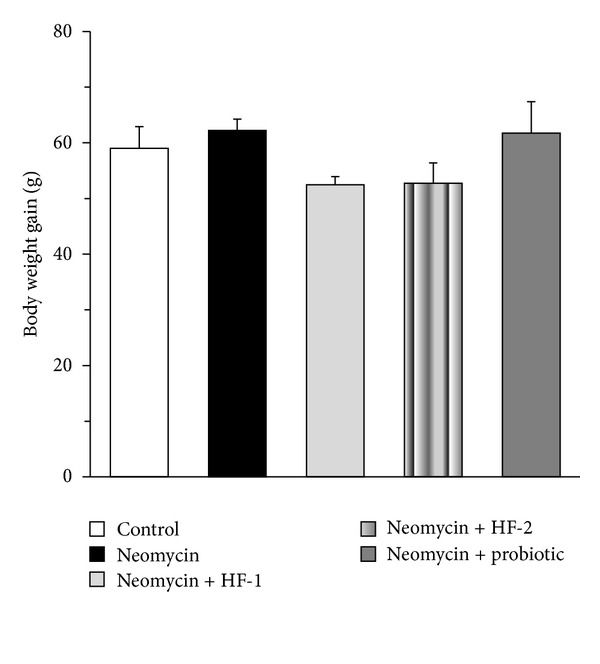
Effect of oral treatment of neomycin either alone or in combination with herbal formulation 1 (HF-1), herbal formulation 2 (HF-2), and probiotic on the body weight changes of rats. The detailed treatment regimen and experimental conditions are described in Section 2. Values are means ± SEM, *n* = 4. No significant difference in the body weight gain of the animals was found between the experimental groups.

Treatment with neomycin did not produce any significant change in the body weight gain of the rats (Figure 2). Exposure to HF-1, or HF-2 as well as the probiotics also did not affect the body weight gain of the neomycin-treated animals. 

### 3.3. Effect of Neomycin Either Alone or in Combination with HF-1, HF-2, and Probiotic on the Water and Relative DNA Content of *Lactobacillus* spp. in the Stool of Animals

**Figure 3:**
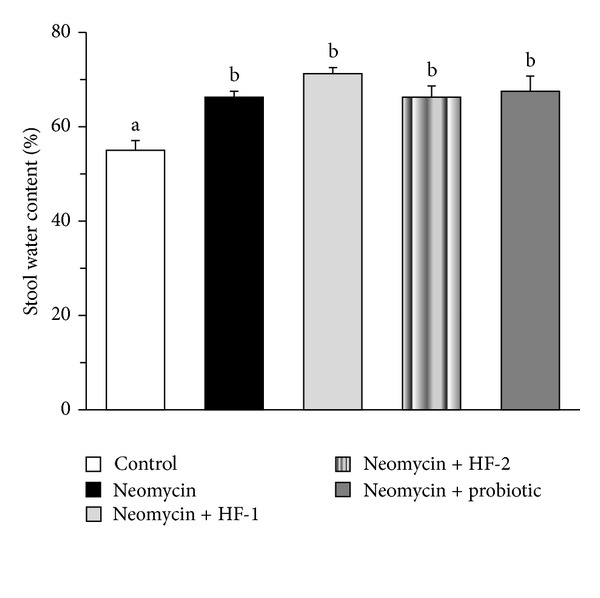
Effect of oral treatment of neomycin either alone or in combination with herbal formulation 1 (HF-1), herbal formulation 2 (HF-2), and probiotic on the fecal water content of rats (expressed as % of wet weight). The detailed treatment regimen and experimental conditions are described in Section 2. Values are means ± SEM, *n* = 4. Means without a common letter differ, *P* < 0.05.

Almost all antibiotic treatments may cause a range of clinical symptoms, most commonly diarrhea also known as antibiotic-associated diarrhea (ADD). There are a number of possible mechanisms by which antibiotics can induce AAD such as destabilization of the composition and function of the normal intestinal microflora, overgrowth of pathogenic microbes like *Clostridium difficile* and their toxin production, and allergic and toxic effects of antibiotics *per se* on the intestinal mucosa or their pharmacological effects on motility [36]. In our study, physical examination of the stool revealed the onset of semisolid appearance of the faeces of animals in between day 2 and day 3 of neomycin treatment, which continued to the end of the study period (data not shown). Consistently, a significant augmentation in the stool water content was recorded in the neomycin-treated rats as compared to control at the end of study (Figure 3). The above two assessments thus indicate the onset of diarrhea in the animals in response to neomycin treatment.

**Figure 4:**
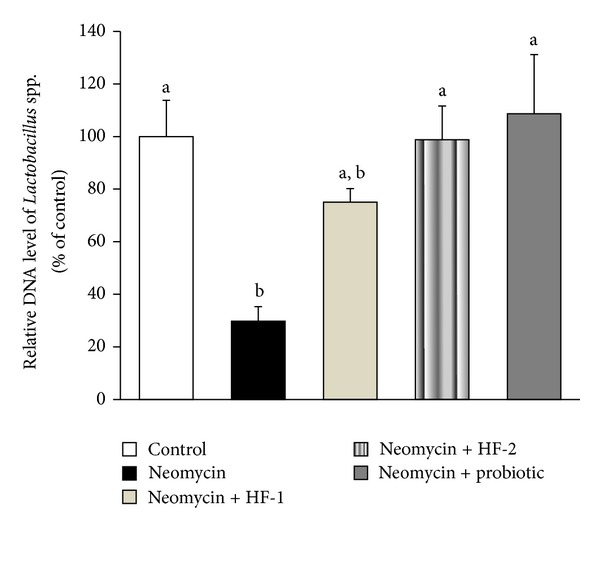
Effect of oral treatment of neomycin either alone or in combination with herbal formulation 1 (HF-1), herbal formulation 2 (HF-2), and probiotic on the content of DNA (gene encoding 16S rRNA) of *Lactobacillus* spp. in relation to that of universal bacteria in the stool of rats. The detailed treatment regimen and experimental conditions are described in Section 2. The relative DNA content of *Lactobacillus* spp. in the stool of control group was set to 100%. Values are means ± SEM, *n* = 4. Means without a common letter differ, *P* < 0.05.

The estimation of fecal DNA content of *Lactobacillus* spp. in relation to that of universal bacteria as an indirect measure of the abundance of *Lactobacillus* spp. in the stool was significantly depleted (70% reduction) in neomycin-treated rats as compared to that of control (Figure 4). This is in parallel with an earlier report where oral administration of neomycin resulted in the depletion of aerobic intestinal bacterial counts [6]. Thus, our results are suggestive of the destabilization of the normal microbial environment of the GIT by neomycin that could eventually lead to the onset of diarrhea. As expected, complete restoration of the fecal population of *Lactobacillus* spp. was seen in the neomycin-treated animals (264% increase), when they were cotreated with probiotic containing *L. acidophilus*. Notably, the fecal *Lactobacillus* spp. content of neomycin-treated rats also increased significantly (231%) to almost the control level when they were cotreated with HF-2. In contrast, cotreatment with HF-1 produced a marked but insignificant increase in fecal population of *Lactobacillus* spp. in neomycin-treated rats. These results are indicative of the beneficial impact of fermentation/refermentation of the herbs and the probiotic strains being involved in this process (*L. mesenteroides* and *B. licheniformis*) on the GIT of neomycin-treated rats. It is conceivable that a net increment in the polyphenol content of the mixed herbal formulation as a consequence of prior fermentation of the component herbs (Figure 1) may account for one of the possible explanations of the above fact. It has been found that polyphenols can alter the gut microecology and may confer positive gut health benefits by affecting the total number of beneficial microflora in the gut [37]. Additionally, the enzyme dextransucrase (EC 2.4.1.5) produced by *Leuconostoc *spp., the bacterial strain used in our study for fermentation, plays a key role in the formation of a number of oligosaccharides or dextran polymers. These polymers could act as prebiotics [38], which selectively promote the growth of some beneficial bacterial species (e.g., *Lactobacilli*, *Bifidobacteria*) and thereby equilibrate the intestinal microflora [39]. Besides, it has been found that the *Bacillus* spp., which was also used in our study for fermentation, facilitates the growth of *Lactobacillus* murinus in mice under specific dietary conditions [40]. 

However, despite of the above fact, no significant difference in the stool water content was seen between the neomycin-treated rats and the animals treated with neomycin in combination with HF-1, HF-2, or probiotic (Figure 3). This suggests that the beneficial effect of HF-2 or probiotic on gut was not directed against neomycin-induced diarrhea. Notably, in a clinical study despite their proven anti-diarrheal activities, kaolin-pectin and lomotil failed to exert any drug effect on the stool water content of subjects suffering from acute diarrhea [41].

### 3.4. Impact of Neomycin Either Alone or in Combination with HF-1, HF-2, and Probiotic on the Intestinal Histology As Well As Gene Expression of Key Inflammatory Mediators

**Figure 5:**
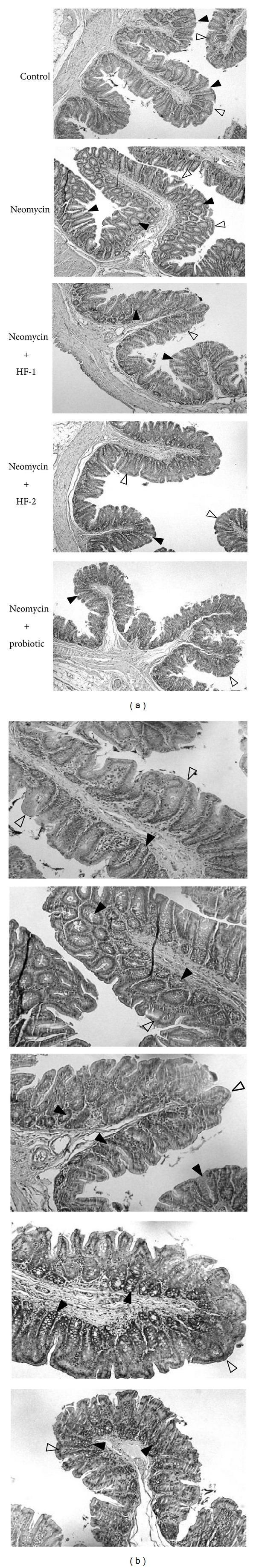
Representative microscopic images of hematoxylin- and eosin-stained cecal tissue sections of rats in different experimental groups. The detailed treatment regimen and experimental conditions are described in Section 2. Column (a): original magnification ×10; Column (b): an enlarged projection (original magnification ×20) of a selected portion of the tissue section represented by (a). The hollow and solid arrowhead represents the mucus layer and mucosal crypts, respectively. The tissue sections of control animals show normal histological architecture of the mucosa characterized by the presence of distinct and intact mucus layer and the regular appearance of deep crypts that open to the surface epithelium. In contrast, the cecal mucosa of the neomycin-treated rats demonstrate an impaired structure encompassing frequent disruption of the mucus layer, abrupt reduction in the number of deep crypts as well as a marked abundance of smaller and aberrant crypts that are dispersed in multilayers. The administration of both herbal formulations as well as the probiotic in the neomycin-treated rats tends to restore the normal architecture of the cecum but with varied degrees.

**Figure 6:**
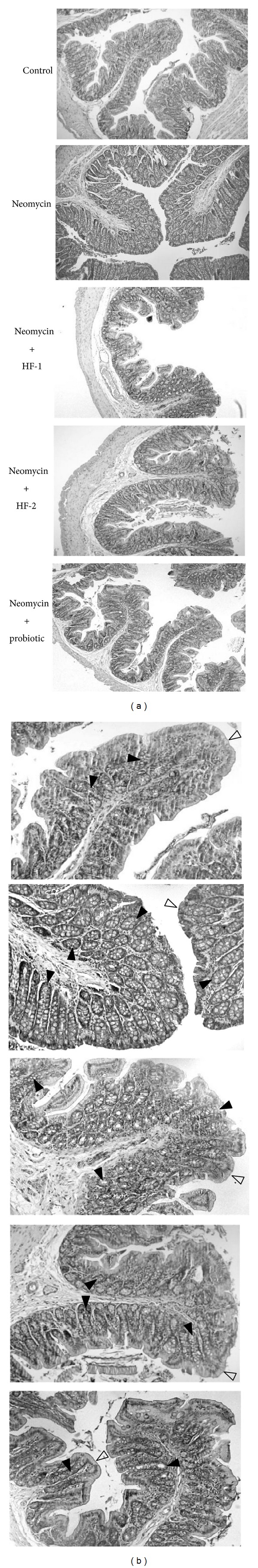
Representative microscopic images of hematoxylin- and eosin-stained colonic tissue sections of rats in different experimental groups. The detailed treatment regimen and experimental conditions are described in Section 2. Column (a): original magnification ×10; Column (b): an enlarged projection (original magnification ×20) of a selected portion of the tissue section represented by (a). The hollow and solid arrowhead represents the mucus layer and crypts, respectively. The tissue sections of control animals show normal histological architecture of the mucosa characterized by the presence of distinct and intact mucus layer and the regular appearance of deep crypts that are open to the surface of epithelium. In contrast, the colonic mucosa of the neomycin-treated rats demonstrates an aberrant structure with the following features: a well-defined surface epithelium but with a non-prominent and disrupted outer mucus layer; deprivation of deep crypts that are open to the surface of epithelium; a strikingly high abundance of smaller and aberrant crypts that are dispersed in multilayers. The administration of both herbal formulations as well as the probiotic in the neomycin-treated rats tends to restore the normal architecture of the cecum but with varied degrees.

Histological evaluation of the tissue samples of control rats demonstrated a normal architecture of both cecum and colon (Figures 5 and 6, resp.) with the appearance of a prominent mucus layer. Treatment with neomycin caused a notable disruption in the architecture of both of the tissues with the following overall characteristics: less distinctive and impaired mucus layer, often deformed; reduction in the number of deep crypts that are open to the surface of epithelium; and abundance of smaller and aberrant crypts that are dispersed in multilayers. Earlier studies have shown that neomycin can produce a number of adverse effects on the histology of GIT such as aberration of crypt cells, blunting of villi with irregular outline leading to the alteration in the ratio of villous to nonvillous portions of mucosa, decline in the number of goblet cells, and epithelial cell damage [7–10]. Notably, in our study, both the herbal preparations as well as probiotic ameliorated the neomycin-induced histological disruption of the intestine. This is evident by the presence of a well-defined and non-disrupted mucus layer in both the cecal and colonic mucosa of neomycin + HF-1, neomycin + HF-2, and neomycin + probiotic groups. However, the neomycin + HF-2 group exhibited a more normal structure and organized distribution of the crypts in both cecum and colon than that shown by the neomycin + HF-1 rats. The histological architecture of the intestine of neomycin + probiotics group, on the other hand, was almost similar to that of the control group. The results thus further support the beneficial impact of fermentation/refermentation of the herbs as well as the probiotics on the protection of intestine from neomycin insult.

However, despite the above histological changes made by neomycin, neither the cecum nor the colon of the animals in any of the treatment groups exhibited the signs of inflammation such as edema, hemorrhage, or marked inflammatory cell infiltration in both the lamina propria and submucosa region. In parallel, also no significant alteration in the colonic expression of the key inflammatory mediators as well as anti-inflammatory protein IL-10 was evident in between the experimental groups (Table 4). Collectively, our results suggest that neomycin-induced changes in intestinal histology and its amelioration by HF-1, HF-2, or probiotic are not linked to the inflammatory process, rather than other mechanism(s) that needs further studies to be fully understood.

### 3.5. Impact of Neomycin Either Alone or in Combination with HF-1, HF-2, and Probiotic on the Colonic Expression MUC-2 Gene

Mucin, which is produced by the goblet cells, constitutes the chief protective mucus layer of the GIT. So far, 21 different mucin genes have been identified among which MUC-2 is the most important one in the IM [2]. In our study, no significant difference in the colonic MUC-2 expression was seen between the control and neomycin-treated groups (Table 5). However, the MUC-2 mRNA level in colon was significantly augmented in neomycin + HF-1, neomycin + HF-2, and neomycin + probiotics groups in comparison to both control and neomycin groups, accounting for a 114%, 146%, and 225% increase over the control, respectively. This suggests that enhancement in the transcription of MUC-2 is one of the probable mechanisms through which the above three agents combat neomycin insult on intestine. 

### 3.6. Impact of Neomycin Either Alone or in Combination with HF-1, HF-2, and Probiotic on the Colonic Expression of Tight Junction Proteins and the Serum CRP and IFN-*γ* Level

Microbial colonization of the gut by probiotics confers the protection of the epithelial barrier by maintaining tight junction protein expression and preventing apoptosis upon chemically induced colitis [4]. Accordingly, changing the microbial population through antibiotic treatment could impair the strength of the intestinal epithelial cell (IEC) barrier through alterations in tight junction protein expression [4]. Declined expression of tight junction proteins would augment the permeability of the IEC barrier allowing commensal leakage into the underlying lamina propria [4].

**Figure 7:**
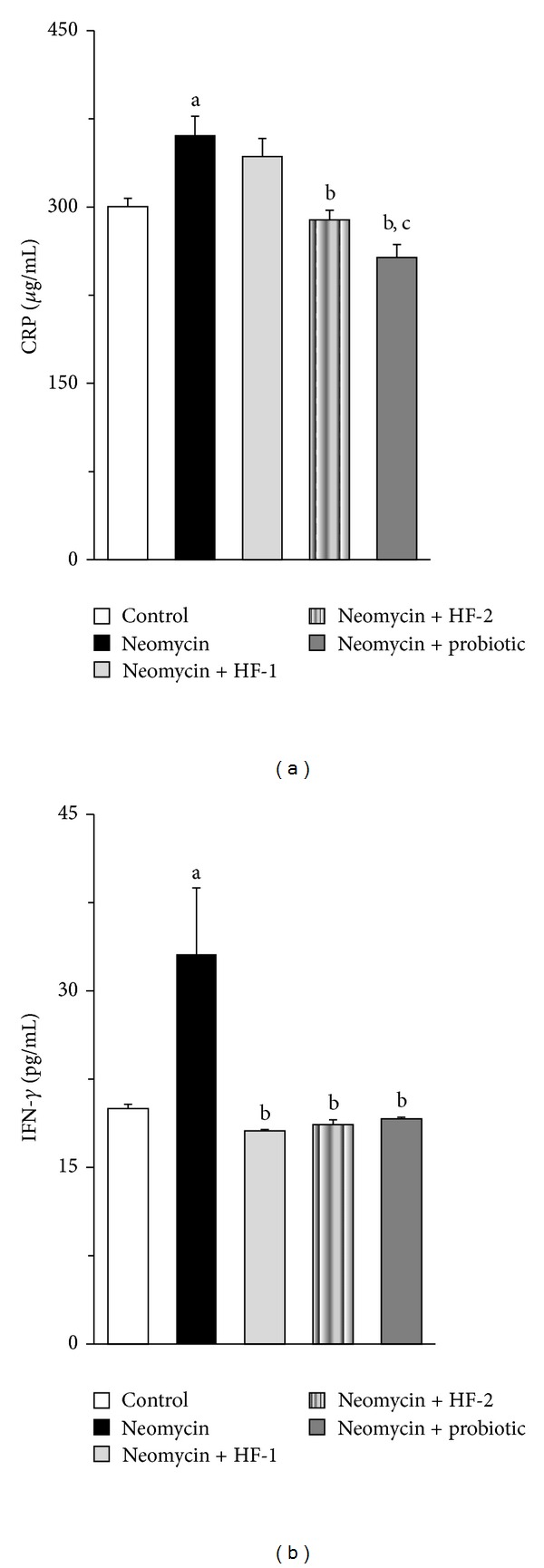
Effect of oral treatment of neomycin either alone or in combination with herbal formulation 1 (HF-1), herbal formulation 2 (HF-2), and probiotic on the serum CRP (a) and IFN-*γ* (b) levels in rats. The detailed treatment regimen and experimental conditions are described in Section 2. Values are means ± SEM, *n* = 4. In case of IFN-*γ*, data were log-transformed prior to analysis by ANOVA. ^a^Different from control group. ^b^Different from neomycin-treated group. ^c^Different from neomycin + HF-1 group, *P* < 0.05.

In keeping with the above, in our study, a decline in the gut *Lactobacillus* spp. by neomycin treatment was associated with a significant reduction in the colonic expression of tight junction proteins ZO-1 (35% decline) and claudin-1 (27% reduction) (Table 5), indicating the possibility of impaired intestinal barrier function as a consequence of this antibiotic treatment. This in turn can augment intestinal permeability [4], which may promote the translocation of viable indigenous microbes from the GIT to extraintestinal sites as found in mice in response to the oral treatment of penicillin, metronidazole, or clindamycin [42]. Indeed, antibiotic-mediated perturbations of the intestinal microflora could alter the host susceptibility to enteric infection [11]. Taking the above into consideration, in our study, the possibility of bacterial translocation could not be excluded in the neomycin-treated rats since they exhibited significantly augmented levels of CRP (20%) and IFN-*γ* (66%) in the serum as compared to control (Figures 7(a) and 7(b)), and the elevation of these two proteins is associated with the state of infection or disease in addition to other factors [43, 44]. Coadministration of HF-1 in neomycin-treated rats increased the ZO-1 transcription insignificantly (*P* = 0.054) but almost to the control level and augmented the claudin-1 expression insignificantly but to a level which did not differ significantly from that of control (*P* = 0.218). On the other hand, the expression of ZO-1 and claudin-1 in the neomycin + HF-2 and neomycin + probiotic groups was significantly higher than that of the neomycin-treated group. In keeping with the above profile, cotreatment with HF-2 or probiotic significantly depleted the serum content of both CRP, and IFN-*γ* in the neomycin-treated rats (Figures 7(a) and 7(b)). While the level of serum IFN-*γ*, but not the CRP was reduced significantly in neomycin + HF-1 group in comparison to that of neomycin group alone (Figures 7(a) and 7(b)). Taken all these into consideration, it is conceivable that the mixed herbal preparation exerts beneficial impact on the intestinal barrier function of neomycin-treated rats and this property is further potentiated upon fermentation/re-fermentation of the individual extract of the formulation along with the use of the probiotics being employed for fermentation.

Our study has some limitations. First, we have selected *Lactobacillus* spp. as the only representative bacterial strain to evaluate the impact of neomycin treatment on the intestinal microbial community. The rationale for this selection is based on the fact that the members of *Lactobacillus* spp. represent a vital part of the healthy human intestinal flora. Through the production of vitamins and enzymes, *Lactobacillus* spp. can affect the metabolism of a host [45, 46], and via the production of antimicrobial compounds, Lactobacilli may exert beneficial impact by preventing the proliferation of undesired pathogens [47–49]. Application of antibiotics can destabilize the indigenous intestinal flora, leading to a significant decrease in *Lactobacillus* spp. [50–52], which is a common problem in treatment of infectious diseases and postoperative septic complications [53]. An individual with a depleted indigenous flora is more susceptible to secondary infections and overgrowth of undesired microorganisms, leading to diarrhea and even pseudomembranous colitis and development of distant organ failure [53–55]. Restoration of the human indigenous intestinal flora in diarrheic condition through the administration of *Lactobacillus* spp. has been tried in several studies, mostly with positive results [52, 56–58]. Besides, it has been shown that the member of *Lactobacillus* spp. can improve intestinal barrier function by affecting the expression of genes in the tight junction (TJ) signaling pathway in healthy intestinal epithelial cells, in particular the genes encoding occludin and its associated plaque proteins, ZO-1, ZO-2, and cingulin [59]. However, in addition to *Lactobacillus* spp., other probiotics such as *Bifidobacterium* spp., *Enterococcus faecium*, and *Streptococcus boulardii* play a vital role in the protection of intestine against antibiotic-associated diarrhea [60]. Therefore, the impact of neomycin on the above mentioned gut probiotics should also be evaluated in future studies in order to further understand the molecular mechanism of neomycin-mediated insult on intestine.

Second, the present study did not identify the key components of the herbal formulations that are acting against neomycin insult. Notably, previous studies have identified a number of gastroprotective compounds from the same herbs used in our herbal formulations. In one study, it has been found that, among five sesquiterpenoids (atractylon, atractylenolide-I (AT-I), AT-II, AT-III, and biatractylolide) isolated from Bai Zhu, AT-III is the principal gastroprotective component in ethanol-induced gastric mucosal damage in *in vitro* and *in vivo* models [61]. The gastroprotective action of AT-III was shown to be mediated via inhibition of matrix-metalloproteinase-(MMP-) 2 and MMP-9 expression, decreasing the extracellular matrix damage and preventing gastric ulcer formation [61]. In another study, AT-II was found to be one of the principal constituents of Tong-Xie-Yao-Fang (a famous traditional Chinese formula containing ARM as one of the ingredients) which has been widely used for clinical treatment of diarrhea-predominant irritable bowel syndrome in China [62]. On the other hand, the flavonoid genistein which is present in Dolichoris Semen has been shown to protect intestinal TJ barrier function against oxidative stress, acetaldehyde, enteric bacteria, and inflammatory cytokines [63]. More specifically, genistein blocks the tyrosine phosphorylation of the TJ proteins induced by oxidative stress and acetaldehyde, which leads to the disassembly of the proteins from the junctional complex [63]. Based on the above information, it is conceivable that future in-depth studies will be needed to identify the active components of our herbal formulations that are operating against neomycin insult. 

Third, our study did not elucidate the exact fermentation-mediated chemical changes in the HF-2 formulation which improved the pharmacological activities of the herbs. It is conceivable that fermentation-mediated augmentation in polyphenol content might be a contributing factor for HF-2 to exert beneficial impact on gut. However, in this context, the possibilities of involvement of other fermentation-derived and/or -modified chemical substances of HF-2 formulation in the intestinal protection against neomycin insult should also be thoroughly investigated. Finally, future studies should also be conducted to evaluate whether the probiotics used in our experiment for the fermentation of the herbs could play any role in the protection of intestine against the adverse effect of neomycin, and if so, further investigations would be needed to understand the mechanism behind this. 

## 4. Conclusions

In summary, our results reveal the protective role of a formulation containing extracts of three dietary herbs against the neomycin-induced adverse effects on the intestine of rats, which is driven through a number of mechanisms and which is potentiated upon fermentation/refermentation in association with the probiotics employed. Further studies are needed to identify the compound(s) and mediator(s) in the proposed herbal formulations that are responsible for conferring the protective effects against antibiotic-induced intestinal disorder.

## Figures and Tables

**Table 1:** The procedure of herbal extraction and fermentation in the preparation of HF-1 and HF-2 formulations.

Formulation	Herbs	Herbal extraction	Processing of herbal extract			
			Reconstitution of dried extract	Dispersion of extract suspension	Supplementation of extract suspension	Fermentation of extract
HF-1	ARM	Decoction followed by drying of extract	10% (w/v) suspension in water	Ultrasonication and shaking at 70°C	Glucose (2% w/v)	Unfermented
	MMF	Decoction followed by drying of extract	10% (w/v) in water	Ultrasonication and shaking at 70°C	Glucose (2% w/v)	Unfermented
	DS	Decoction followed by drying of extract	10% (w/v) in water	Ultrasonication and shaking at 70°C	LB broth powder (2.5% w/v)	Unfermented

HF-2	ARM	Decoction followed by drying of extract	10% (w/v) in water	Ultrasonication and shaking at 70°C	Glucose (2% w/v)	Fermented by *Bacillus licheniformis *
	MMF	Decoction followed by drying of extract	10% (w/v) in water	Ultrasonication and shaking at 70°C	Glucose (2% w/v)	Fermented by *Leuconostoc mesenteroides *
	DS	Decoction followed by drying of extract	10% (w/v) in water	Ultrasonication and shaking at 70°C	LB broth powder (2.5% w/v)	Fermented by *Bacillus licheniformis *

**Table 2:** The sequences of the primers employed in qRT-PCR analysis of rat stool bacterial DNA targeting 16S rRNA gene of the universal bacteria or *Lactobacillus* spp.

Target gene	PS	Sequence (5′–3′)	OAT	References
Universal bacteria	F	CCTACGGGAGGCAGCAG	60°C	[17]
	R	ATTACCGCGGTGCTGG		

*Lactobacillus* spp.	F	GAGGCAGCAGTAGGGAATCTTC	60°C	[18]
	R	GGCCAGTTACTACCTCTATCCTTCTTC		

PS: primer sets; F: forward; R: reverse; OAT: optimized annealing temperature.

**Table 3:** Primer sequences used for the detection of colonic expression of key inflammatory mediators and cytokines as well as tight junction proteins and MUC-2 in rats using qRT-PCR.

Target gene	PS	Sequence (5′–3′)	OAT	References
Claudin-1	F	TGTAATTTCAGGTCTGGCGACA	53°C	[19]
	R	GGATAAGGCCGTGGTGTTGG		

COX-2	F	CTCTGCGATGCTCTTCCGAG	48°C	[20]
	R	AAGGATTTGCTGCATGGCTG		

GAPDH	F	ATGGCACAGTCAAGGCTGAGA	53°C	[21]
	R	CGCTCCTGGAAGATGGTGAT		

ICAM-1	F	CGTGGCGTCCATTTACACCT	58°C	[21]
	R	TTAGGGCCTCCTCCTGAGC		

IL-1*β*	F	CACCTCTCAAGCAGAGCACAG	53°C	[22]
	R	GGGTTCCATGGTGAAGTCAAC		

IL-6	F	GCCCTTCAGGAACAGCTATGA	55°C	[20]
	R	TGTCAACAACATCAGTCCCAAGA		

IL-10	F	TGCAACAGCTCAGCGCA	53°C	[23]
	R	GTCACAGCTTTCGAGAGACTGGAA		

Occludin	F	TTACGGCTATGGAGGGTACAC	50°C	[24]
	R	TGACGCTGGTAACAAAGATCAC		

MUC-2	F	GCCAGATCCCGAAACCA	50°C	[25]
	R	TATAGGAGTCTCGGCAGTCA		

TNF-*α*	F	GGTGATCGGTCCCAACAAGGA	45°C	[26]
	R	CACGCTGGCTCAGCCACTC		

ZO-1	F	TTCCGCCTCTGTCCAACTCT	53°C	[24]
	R	ATGGGGGTGGGTCTGGTTTC		

PS: primer sets; F: forward; R: reverse; OAT: optimized annealing temperature.

**Table 4:** Effect of oral treatment of neomycin either alone or in combination with HF-1, HF-2, and probiotic on the colonic expression of key inflammatory mediators and cytokines in rats^1^.

Treatment	Level of mRNA (% of control)					
	COX-2	TNF-*α* ^2^	IL-1*β* ^>2^	IL-6	IL-10	ICAM-1
Control	100.00 ± 15.29	100.00 ± 15.04	100.00 ± 3.91	100.00 ± 1.67	100.00 ± 3.60	100.00 ± 4.08
Neomycin	91.09 ± 7.01	86.02 ± 4.24	107.55 ± 2.29	103.86 ± 3.95	105.55 ± 5.00	97.55 ± 3.22
Neomycin + HF-1	86.99 ± 10.94	108.39 ± 1.59	109.21 ±1.35	104.37 ± 9.31	104.14 ± 3.82	94.38 ± 1.61
Neomycin + HF-2	102.55 ± 2.47	104.48 ± 1.99	105.74 ± 2.56	103.82 ± 2.53	116.05 ± 5.29	92.75 ± 2.00
Neomycin + probiotic	101.86 ± 7.48	111.43 ± 3.55	108.91 ± 7.44	103.70 ± 5.26	116.30 ± 5.47	97.97 ±3.25

^1^The detailed treatment regimen and experimental conditions are described in Section 2. The level of expression of genes in control group was set to 100%. Data are means ± SEM, *n* = 4. ^2^Data were log-transformed prior to analysis by ANOVA. None of the genes showed significant difference in expression between the groups.

**Table 5:** Effect of oral treatment of neomycin either alone or in combination with herbal formulation 1 (HF-1), herbal formulation 2 (HF-2), and probiotic on the colonic expression of key tight junction proteins and MUC-2 in rats^1^.

Treatment	Level of mRNA (% of control)			
	ZO-1	Claudin-1	Occludin	MUC-2^2^
Control	100.00 ± 5.70^a^	100.00 ± 7.71^a,b^	100.00 ± 4.08^a^	100.00 ± 17.29^a^
Neomycin (Neo)	65.11 ± 5.90^b^	72.87 ± 3.12^c^	97.11 ± 1.47^a^	98.03 ± 12.16^a^
Neo + HF-1	98.04 ± 8.43^a,b^	80.77 ± 5.24^a,c^	99.85 ± 2.60^a^	213.73 ± 13.40^b^
Neo + HF-2	109.38 ± 9.46^a^	98.05 ± 3.68^a,d^	101.35 ± 8.47^a^	246.28 ± 13.41^b^
Neo + probiotic	115.00 ± 5.37^a^	113.98 ± 5.57^b,d^	110.40 ± 5.70^a^	325.29 ± 87.25^b^

^1^The detailed treatment regimen and experimental conditions are described in Section 2. The level of expression of genes in control group was set to 100%. Data are means ± SEM, *n* = 4. ^2^Data were log-transformed prior to analysis by ANOVA. Means in a column with superscripts without a common letter differ, *P* < 0.05.
